# Black-Silicon on Micropillars with Minimal Surface Area Enlargement to Enhance the Performance of Silicon Solar Cells

**DOI:** 10.1186/s11671-016-1716-y

**Published:** 2016-11-07

**Authors:** Jiann Shieh, Chengyun You, Chiachen Chiu, Jianming Liu, Pingyu Shih

**Affiliations:** Department of Materials Science and Engineering, National United University, Miaoli, 36003 Taiwan, Republic of China

**Keywords:** Black silicon, Micropillar, Surface area, Reflection, Solar cell

## Abstract

Although black silicon is used widely as an antireflection coating in solar cells, the corresponding electrical properties are usually poor because the accompanied enlarged surface area can result in increased recombination. Moreover, the high aspect ratio of fragile nanostructured black silicon makes conformal passivation even more challenging. Micropillars are promising alternative candidates for efficiently collecting carriers because the diffusion distance for minority carriers to reach the p–n junction can be shortened; however, the pillar diameter is usually larger than the wavelength of light, inherently increasing the surface reflection. In this paper, we report an approach for decreasing the surface reflection of black silicon and micropillar structures: combining them together to create a dual-scale superstructure that improves the electrical and optical properties concurrently. The reflection of the micropillars decreased significantly as the surface was decorated with a thin black silicon layer, and the thickness of black silicon required for low reflection was reduced as the black silicon was positioned atop micropillars. Three-dimensional finite difference time domain simulations supported these results. Moreover, with such a thin decoration layer, the superstructure displayed improved power conversion efficiency after silicon nitride passivation, suggesting great potential for such superstructures when applied in solar cells.

## Background

A low surface reflection is essential for the trapping of incident light in high-performance silicon solar cells [1, 2]. Exploiting a graded change in refractive index between air and silicon, several nanostructures, including moth-eye-like nanotips, have been applied to fabricate “black silicon” (b-Si) [3–5]. This approach through structural modulation can decrease the surface reflection of materials efficiently over broad spectral ranges. For example, the average total reflection of amorphous silicon thin films over the wavelength range 300–800 nm can decrease from approximately 48 % to less than 1 % after plasma etching [6]. Other optical properties of silicon-based materials have been widely investigated [7, 8], but the application of b-Si in solar cells also requires consideration of its electrical properties. Typically, the performance of b-Si solar cells is not superior to that of the traditional solar cells having antireflective pyramid surface structures, even though the surface reflection of b-Si is relatively lower [9, 10]; as a result, high-quality conformal passivation is usually required for nanostructured b-Si [11, 12]. The combination of the nanostructures’ high surface area and high density of trapping sites has been suggested to account for the poor electrical properties [9–12]. Accordingly, simultaneously improving both the electrical and optical properties of b-Si remains a challenge.

Micropillar structures are possible candidate materials for enhancing the performance of planar solar cells [13–15]. With a radial junction profile, the distance required for the minority carrier to reach the junction is decreased, increasing the efficiency of carrier collection. In addition, the pillar profile has the attractive feature of low reflection through the multiple scattering [16], photonic crystal [17], or Mie resonance mechanism [18]. Nevertheless, small micropillars (e.g., nanopillars, nanowires) encounter a problem—large surface area—similar to that of b-Si; therefore, large-radius micropillars are more likely to enhance the electrical properties when taking surface area into consideration. Extensive research on microwires and micropillars with radial junctions has revealed that a pillar radius on the scale of the minority-carrier diffusion length (which depends on the wafer quality, but can be several micrometers) results in the collection of more carriers excited within the micropillars [19, 20]. Unfortunately, surface reflection inevitably increases for a larger pillar diameter, especially when the diameter is greater than the wavelength.

To overcome the trade-off between the optical and electrical properties, nanoparticle scattering [21] and asymmetric profile design [22] have been proposed to enhance light trapping without significantly increasing the surface area. Furthermore, a hierarchical structure combining micropyramids and nanowires has been reported for enhanced light-trapping [23, 24]. Nevertheless, little is known about decreasing the reflection of micropillars or that of low-reflective b-Si having a low surface area. In a previous study, we found that two nanostructures could mutually enhance their antireflection properties when integrated together [25]. We have also presented the initial idea of a dual-scale superstructure, combining b-Si and micropillars, for solar cells and demonstrate that lower reflection and higher photoelectronic conversion can both be obtained at the same time [26]. Herein, we calculated the surface area enlargement from b-Si, micropillar as well as superstructure, and revealed that b-Si was accompanied by high surface area. When the b-Si was fabricated atop the micropillars to form a superstructure, the surface area enlargement can be inhibited for lower reflection. We also performed finite difference time domain (FDTD) simulation to support the results of low reflection from superstructure. As a result, the power conversion efficiency (PCE) of devices incorporating the b-Si and micropillar structures improved when combining them together, especially when the thin b-Si layer was passivated using a silicon nitride layer.

## Methods

The b-Si was prepared through metal-assisted chemical etching (MACE), where the silicon was oxidized first and then the oxide was etched away, so that the surface damage on the nanostructured silicon could be inhibited [27–30]. For MACE, an aqueous solution containing 5 M HF and 10^–3^ M AgNO_3_ was placed on the samples to reduce Ag ions to form Ag nanoparticles (NPs) by acquiring electrons from silicon; the silicon beneath the Ag NPs was selectively oxidized by hole injection from the metal. The oxide was then etched away by applying a drop of HF (5 M) and H_2_O_2_ (0.1 M) for 1–10 s, such that the Ag NPs sank into the silicon to form pits on the surfaces. Accordingly, the particle density and the porous profile could be controlled by varying the AgNO_3_ concentration, while the etching depth was proportional to the etching time. The residual Ag NPs were removed using HNO_3_ solution, and then the samples were washed with DI water and dried under a N_2_ flow.

The micropillars were patterned using i-line lithography and etched by Cl_2_/HBr plasma on a 6-in p-type Si wafer (1–10 Ω cm; 675 μm). The radial p–n junction was prepared by implantation (energy 36 keV; dose 5 × 10^14^ cm^–2^) as the wafer was tilted by 12° off the major plane with four rotations (0°, 90°, 180°, 270°) to increase the doping uniformity. The junction depths on the top surfaces and side walls were approximately 200 and 50 nm, respectively [15]; such thin structures led to less recombination in the heavily doped region. A 1-μm aluminum back-side electrode was coated by e-beam evaporation and annealed at 600 °C under H_2_ ambient for 10 min to improve the contact between Si and Al. Plasma-enhanced chemical vapor deposition (PECVD) was performed at 300 °C to deposit 75-nm silicon nitride as a passivation layer. A baked Ag paste was used as the front-side electrode, which was positioned outside of the micropillar array region.

To prepare a superstructure device, the native oxide on the micropillars was first removed using dilute HF solution, followed by applying the MACE process to create b-Si on the surface of the micropillar array. The process was performed by dropping the etchant on the array to prevent etching on the backside of the silicon wafer. The doping process for all the devices was performed prior to b-Si formation, thereby ensuring a more uniform doping profile and a shallower doping layer and, thus, higher PCE [31].

The morphologies of samples were characterized using scanning electron microscopy (SEM, JEOL-6700F). The contour plot of b-Si was sketched out using ImageJ software. The total reflections in the wavelength range 400–1000 nm were measured using a spectrometer equipped with an integrating sphere to detect all the reflections from the surface. FDTD simulations of the interactions between light and the materials were performed using a commercial program (FDTD solutions, Version 8, Lumerical Solutions). In the simulations, periodic boundaries were applied along the x- and y-boundaries, and perfect matched layers were applied as top and bottom boundaries of the FDTD unit cell. A plane wave light source was set 100 nm above the micropillars; a power monitor was set 100 nm above the light source to detect reflections. Simulated reflection spectra over a broad range of wavelengths were obtained by Fourier transform from the power monitor. Energy conversion measurements were performed under 1-sun illumination using a four-wire connection to eliminate the effects of the lead resistance. The exposed area (16 mm^2^) was defined by an opaque mask. The probes penetrated into the mask and contacted with Ag paste that was printed on the flat surface to decrease the shadowing effect from tips on the exposed region.

## Results and Discussion

**Fig. 1:**
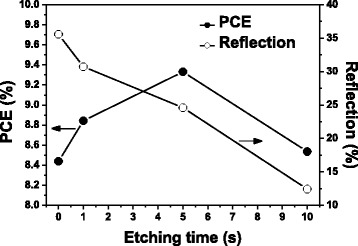
Reflection and PCE of b-Si, plotted with respect to etching time

First, we investigated the effects of the etching conditions on the optical and electrical properties of b-Si. Figure 1 presents the reflection and device PCE plotted with respect to the etching time. The reflection decreased after longer etching times. The PCE, however, increased initially before reaching a maximum and decreasing thereafter, indicating that an enlarged surface area arising from a higher-aspect-ratio profile may degrade the PCE performance after long etching times.

**Fig. 2:**
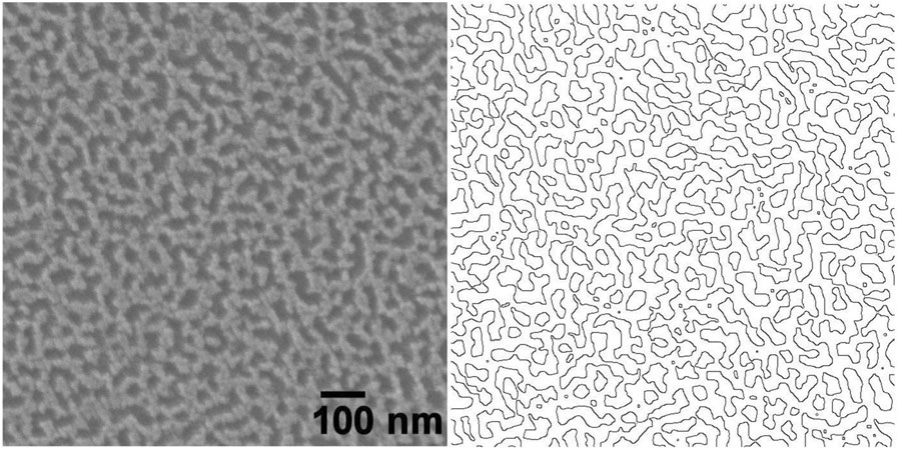
Top-view SEM image of b-Si and corresponding contour plot

To evaluate the degree to which the surface area increased as a result of the b-Si morphology, we estimated the surface area ratio (*R*) between b-Si and flat surface using the Eq. (1)

1 $$ \boldsymbol{R}=\frac{\boldsymbol{L}\times \boldsymbol{H}+{\boldsymbol{S}}_{\boldsymbol{f}}}{{\boldsymbol{S}}_{\boldsymbol{f}}} $$

where *L* is the contour length, *H* is the etching depth, and *S*_*f*_ is the area of the flat surface. Figure 2 presents a typical top-view SEM image and corresponding contour plot for the surface prepared under 10^–3^ M AgNO_3_ for 5 s. Using ImageJ software, we calculated the value of *L* on this plot to be 61,500 nm, thereby obtaining the following relationship between *R* and *H*:

2 $$ \boldsymbol{R}=\mathbf{6.15}\times {\mathbf{10}}^{-\mathbf{2}}\kern.2em \boldsymbol{H}+1 $$

when the etching depth *H* was 50 nm, the value of *R* was 4.07—thus, MACE had great impact on increasing the surface area. In comparison, the surface area ratio between the micropillars and flat Si was only 3.5, even though the pillar height was much larger (2.5 μm). Because the surface area increased sharply upon increasing the etching depth, a thin b-Si layer would be necessary for good electrical performance.

**Fig. 3:**
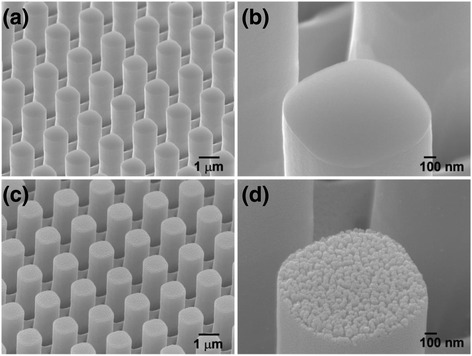
Tilted-view SEM images of **a**, **b** micropillars and **c**, **d** superstructures

Next, we fabricated b-Si on micropillar array to obtain low reflection from a thin b-Si layer. Figure 3a displays an array of micropillars—1 μm × 1 μm square pillars—sitting atop a planar silicon wafer. The pillar height was 2.5 μm, and the spacing between pillars was 1 μm. The enlarged SEM image in Fig. 3b reveals a flat top surface for the micropillars. After MACE with 1 × 10^–3^ M AgNO_3_ for 5 s, nanopores had developed to form b-Si on top of the micropillars, resulting in a dual-scale superstructure (Fig. 3c, d). The cross-sectional SEM image revealed that the depth of these nanopores was approximately 50 nm.

**Fig. 4:**
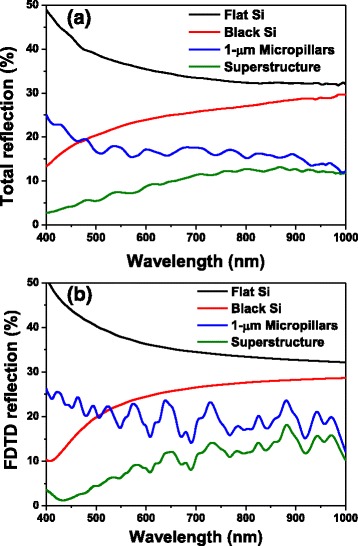
**a** Experimental measurements and **b** FDTD simulations of the reflections from flat Si, b-Si, micropillars, and superstructures

**Fig. 5:**
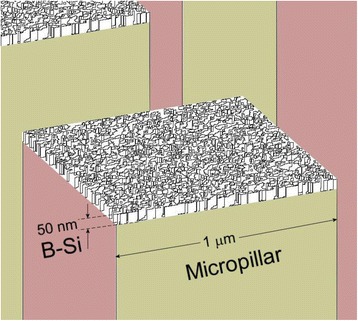
3D model of b-Si sitting atop a micropillar for FDTD simulation

**Fig. 6:**
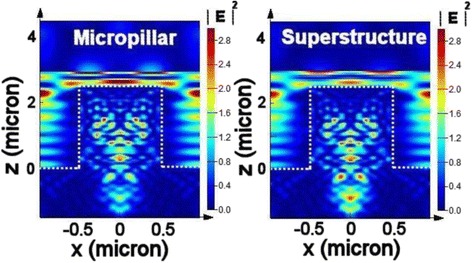
Electric field distributions at 685 nm in a micropillar structure and a superstructure

Figure 4a presents the total reflection from these samples at wavelengths ranging from 400 to 1000 nm; for the micropillar array, it was 16.73 % on average, whereas the superstructure reflected only 9.63 % of the light. Thus, even though the depth of the nanopores was only approximately 2 % of the height of the micropillars, the reflection from the superstructure had decreased to approximately 58 % of that of the micropillars having the flat top surface. The reflection of b-Si, formed from flat silicon after 5 s of MACE, is also presented in Fig. 4a for comparison. Its weighted reflection over the wavelength range 400–1000 nm was 24.6 %, lower than that from the planar silicon (35.3 %), but higher than those from the micropillars and superstructures. To obtain a reflection as low as that from the 5-s etching superstructure, the etching time for b-Si would have to be longer than 10 s, as indicated in Fig. 1. Although b-Si of lower reflection could be obtained using longer etching times, the higher aspect-ratio structures were more fragile and their accompanied high surface areas would limit photoelectronic conversion. We performed FDTD simulations on these samples to verify the experimental observations. In the model, because the etching rate was much faster beneath the Ag nanoparticles, the nanopores of b-Si were aligned vertically; thus, combining the top-view SEM image with the etching depth allowed us to build a three-dimensional (3D) profile of the b-Si layer. We then constructed a model of the superstructure by placing a 3D b-Si on the micropillars (Fig. 5). The results of the simulations were consistent with the experimental results: a thin top layer would decrease the reflection of the micropillars significantly (Fig. 4b). Moreover, Fig. 6 reveals the distribution of the electric field intensity inside and around the pillars, determined through FDTD simulation, as the samples were illuminated under a plane wave having a wavelength of 685 nm; both the micropillar structure and superstructure confined light inside the pillar as a result of resonance [18], with the superstructure reflecting less of the incoming light. In addition, the electric field intensity was higher below the superstructure, suggesting that more incident light could be trapped for passage into the substrate for higher optoelectronic conversion. Although the profiles for the micropillar structure and the superstructure, indicated by the dashed lines, look almost identical, the E-field distribution can be affected significantly by such a thin b-Si surface layer.

**Fig. 7:**
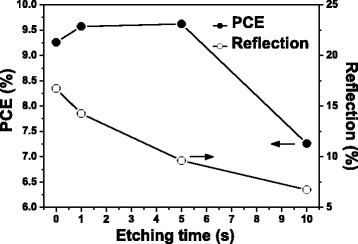
Reflections and PCEs of superstructure devices, plotted with respect to the etching time

Next, we applied the superstructure to silicon solar cells. Figure 7 displays the relationship between the PCE and the etching time for the samples prepared using 10^–3^ M AgNO_3_; similar to the behavior of the planar b-Si surface, the PCE reached a maximum after an appropriate etching duration of 5 s. The corresponding reflections are also presented in Fig. 7. Although reflection from the superstructures had decreased significantly, as discussed in Fig. 4, the PCEs did not improve to such a degree. For example, despite the reflection of the micropillar structure decreasing by 42 % after etching for 5 s, the PCE was enhanced only from 9.26 to 9.62 %. In comparison, when the reflection of the planar silicon had decreased by 30 % upon forming b-Si, the corresponding PCE increased from 8.44 to 9.33 %. Thus, although the superstructure provided much lower reflection, it could not compensate well for the loss in PCE caused by the enlarged surface area. We evaluated the increase in the surface area of the superstructure as we had for the planar b-Si. Compared with the etching on the top surfaces, etching on the side walls of the micropillars was less apparent, as revealed in Fig. 3c, d. Accordingly, we could calculate the value of *R* for the superstructure that was 6.57 times the value for the flat surface (1.88 times the value of *R* of the micropillars). Despite a high value of *R* for the superstructure, the contribution of the b-Si atop the micropillars to the value of *R* was only 3.07, less than that of b-Si atop the planar surface (4.07); thus, although the b-Si fabricated on the micropillars decreased the surface reflection, it did so without increasing the surface area too much.

**Fig. 8:**
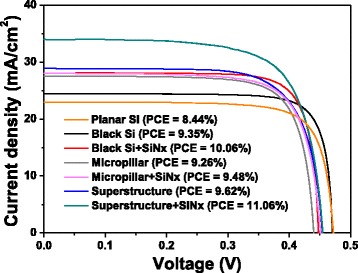
*J*–*V* curves of planar and micropillar devices, prepared with and without MACE and nitride coatings

To retain the attractive properties of the low-reflection superstructure, we deposited 75-nm silicon nitride, through PECVD, as a passivation layer on the superstructures. For the superstructure prepared through treatment with 10^–3^ M AgNO_3_ for 5 s, the photocurrent increased significantly from 28.0 to 33.9 mA cm^–2^, thereby improving the PCE of the superstructure device from 9.65 to 11.02 % (Fig. 8). The *J*–*V* curves of the b-Si and micropillar structures after passivation are presented for comparison. For the micropillars, the nitride coating enhanced the PCE from 9.26 to 9.45 %—a slight improvement. Although the value of *R* was 3.5 for the micropillars, the passivation effect was less significant because the surface was flat (i.e., surface damage may have been less than that in the nanostructure). The PCE of the device prepared using planar b-Si also increased after passivation (from 9.35 to 10.06 %), indicating that the surface area of the nanostructure was well passivated to enhance the PCE. Thus, the passivation of b-Si on the micropillar structures or on the flat plane enhanced the performance in terms of PCE; fabricating b-Si on micropillars is an effective means for passivation because a relatively thin b-Si layer led to lower reflection.

## Conclusions

We have developed an approach, confirmed through experiments and simulations, to significantly decrease the reflections from thin b-Si and micropillar structures by combining them together in the form of a superstructure. Decorating the micropillars with b-Si that were only 2 % in height decreased the reflection by 42 %, while the surface area increased only 1.88-fold relative to the micropillar surface. For b-Si, however, the same etching depth increased the surface area to 4.07 times that of the flat plane. Furthermore, compared with b-Si, this superstructure required a much thinner nanostructure to obtain the same darkness. Applying this superstructure in solar cells led to the PCE of the micropillar device increasing from 9.26 to 9.62 % after the pillar surface had been etched for 5 s with 10^–3^ M AgNO_3_. Applying a nitride passivation layer to the low-reflective superstructure caused the PCE to increase further, to 11.02 %. The slight increase in surface area resulting from the nanostructure in the superstructure presumably contributed to the effective passivation. These results suggest that dual-scale superstructures might facilitate the development of Si solar cells because they provide lower-reflection surfaces with less of an increase in surface area from a thin nanostructure layer.

## Abbreviations

3D: Three dimensional
b-Si: Black silicon
FDTD: Finite difference time domain
MACE: Metal-assisted chemical etching
NP: Nanoparticle
PCE: Power conversion efficiency
PECVD: Plasma-enhanced chemical vapor deposition
SEM: Scanning electron microscopy
